# Association Between Obesity and Microvascular Diseases in Patients With Type 2 Diabetes Mellitus

**DOI:** 10.3389/fendo.2021.719515

**Published:** 2021-10-26

**Authors:** Shan Gao, Hongliang Zhang, Chen Long, Zhenhua Xing

**Affiliations:** ^1^ Department of Geriatrics, The Second Xiangya Hospital, Central South University, Changsha, China; ^2^ Department of Emergency Medicine, Second Xiangya Hospital, Central South University, Changsha, China; ^3^ Emergency Medicine and Difficult Diseases Institute, Central South University, Changsha, China; ^4^ Department of Minimally Invasive Surgery, The Second Xiangya Hospital, Central South University, Changsha, China

**Keywords:** chronic kidney disease progression, neuropathy, retinopathy, type 2 diabetes mellitus, microvascular diseases

## Abstract

This study aimed to evaluate the association between obesity, evaluated by fat mass index (FMI) with the risk of microvascular diseases in patients with type 2 diabetes mellitus (T2DM) and compare the magnitude of associations of FMI, body mass index (BMI), and waist circumference (WC) with the risk of microvascular diseases. We performed a *post-hoc* analysis of the Action to Control Cardiovascular Risk in Diabetes study. The primary microvascular outcomes of the present study included chronic kidney disease (CKD) progression, retinopathy, and neuropathy. Cox proportional-hazards models were performed to evaluate the association of FMI with microvascular diseases. A discordant analysis was performed to compare the magnitude of associations of FMI, BMI, and WC with the risk of microvascular diseases. Our study included 10,251 T2DM participants with a median of 5 years (interquartile range, 4.2–5.7) of follow-up. A total of 6,184 participants developed CKD progression, 896 participants had retinopathy, and 3,213 participants developed neuropathy (Michigan Neuropathy Screening Instrument, >2.0). After the confounding factors were adjusted for, patients in the highest FMI quartile had a higher risk of CKD progression (HR: 1.26, 95%CI: 1.16–1.36) and neuropathy (HR: 1.93, 95% CI: 1.74–2.15), except for retinopathy (HR: 1.17, 95% CI: 0.96–1.43), than those in the lowest quartile. Discordant analyses found that FMI and WC are better in identifying individuals with obesity-related risk of neuropathy, compared with BMI; neither is better in identifying individuals with obesity-related risk of CKD progression and retinopathy. Obesity is associated with CKD progression and neuropathy in T2DM participants. Further randomized trials are needed to test whether obesity control can improve the outcomes of T2DM participants with CKD or neuropathy. FMI and WC are more useful in identifying obesity-related risk of neuropathy compared with BMI in T2DM patients.

**Clinical Trial Registration:**

http://www.clinicaltrials.gov, NCT00000620.

## Introduction

Epidemiological studies show that obesity has become a major problem worldwide and is associated with an increased risk of macrovascular diseases (1, 2). However, the relationship between obesity and microvascular diseases remains controversial, especially in patients with type 2 diabetes mellitus (T2DM) who are at an increased risk of developing microvascular complications (3–6). Prior experimental studies have found that obesity may lead to endothelial dysfunction, while only a small number of epidemiological studies have investigated the association between obesity, evaluated by body mass index (BMI), and microvascular diseases, such as retinopathy, nephropathy, and neuropathy, in patients with T2DM (3, 4, 7, 8).

Most clinical and epidemiological studies mainly use BMI as the preferred measure of obesity or overall adiposity because it is easily measured and highly associated with more objective fat mass (FM) (9, 10). Recent studies have found that BMI is an imperfect indicator of obesity and does not differentiate between lean body mass and FM, which may limit its ability to reveal true health effects (11, 12). FM and lean body mass can vary substantially among individuals with the same BMI (13, 14). However, the relationship between FM and microvascular diseases remains unclear. Therefore, our present study aims to evaluate the association of obesity measured by FM with microvascular diseases. Furthermore, we compared the magnitude of associations of FM, BMI, and WC with the risk of microvascular diseases and found the best indicator of obesity associated with risk of microvascular diseases among patients with T2DM.

## Methods

### Study Design and Population

This study is a *post-hoc* analysis of the Action to Control Cardiovascular Risk in Diabetes (ACCORD) study. Data are available from the Biologic Specimen and Data Repository Information Coordinating Center (BioLINCC). The ACCORD study was a double-parallel treatment trial that included 10,251 T2DM patients from 77 sites in the US and Canada (15). The included patients had a mean age of 62 years, had a mean 10-year history of T2DM, and had a mean glycated hemoglobin A1c level of 8.3%. This trial aimed to assess whether the intensified control of blood glucose, blood pressure, or lipid levels could improve the cardiovascular outcomes of T2DM patients; its design and main study outcomes have been published (15, 16). Intensified glycemic control did not reduce death or nonfatal cardiovascular events but did increase all-cause death (the intensified control of blood glucose was stopped after a mean follow-up of 3.7 years because it increased the risk of all-cause death). Follow-up continued for the remaining participants in the ACCORD trial, with a total follow-up period of 8.8 years. The predefined secondary endpoints of the trial included evaluation of the effects of intensified blood glucose control on the risk of microvascular diseases (*i*.*e*., a composite of nephropathy, retinopathy, and neuropathy). Intensified blood glucose control did not reduce the incidence of advanced measures of microvascular diseases, such as the initiation of dialysis or end-stage renal disease (ESRD); however, it delayed the onset of early microvascular diseases, such as albuminuria and some measures of retinopathy and neuropathy (17). Our present *post-hoc* analysis included both groups (intensive or standard blood glucose treatment), and treatment effect was also used as a confounding factor in our *post-hoc* analysis (17).

### Exposure Variables

The patients were followed every year, at which times data on vital signs including weight and waist circumference were collected. FM was calculated using the prediction equations developed by Lee and his colleagues (18). The prediction equations, which are widely used in epidemiologic studies, showed a high prediction accuracy for FM [*R* (2) = 0.90, standard error of the estimate (SEE) = 2.6 kg for men; *R* (2) = 0.93, SEE = 2.60 kg for women] (12, 19). The prediction equations for FM are presented in **Supplementary Table S1**. The fat mass index (FMI) was calculated as the FM in kilograms divided by height in meters squared. Furthermore, the FMI was used as the primary indicator of obesity, whereas BMI, WC, and W-t-H were employed to verify the associations between obesity and microvascular outcomes Exposures of interest, including FMI, BMI, WC, and W-t-H, were used as cumulative averages of repeated measures during the follow-up period.

### Microvascular Outcomes

In the ACCORD trial, measures of kidney function, diabetic eye complications, and peripheral neuropathy were used to evaluate microvascular disease occurrence. The primary microvascular outcomes of the present study included (1) CKD progression [defined as the doubling of baseline serum creatinine or greater than 20 ml/min per 1.73 m² decrease in the estimated glomerular filtration rate (GFR)], (2) retinopathy (retinopathy needing retinal photocoagulation or vitrectomy), or (3) neuropathy [a score >2.0 on the Michigan Neuropathy Screening Instrument (MNSI) (17)]. The MNSI comprises a structured examination of the feet to identify deformities, dry skin, calluses, infection, fissure, or ulcers as well as evaluation of ankle reflexes and vibration sensation in the great toe. The assessment frequency of CKD progression was every 4 months; retinopathy and neuropathy were evaluated every year. Other complications of kidney function (microalbuminuria, macroalbuminuria, and renal failure), diabetic eye complications (eye surgery for cataract extraction, three-line change in visual acuity, and severe vision loss), and peripheral neuropathy (new loss of either vibratory sensation or ankle jerk during Jendrassik maneuver or sensation to light touch) were predefined as secondary endpoints. The primary and secondary endpoints of our study, diagnostic criteria, and assessment frequency are presented in **Table 1** (17). For each participant and endpoint, observation was censored at the last surveillance time if no endpoint was recorded. If an endpoint was reported, an event time was assigned, with the midpoint between the time of outcome discovery and the most recent previous surveillance time (17).

**Table 1:** Primary and secondary endpoints and assessment frequency.

Primary endpoints	Assessment frequency
CKD progression (doubling of baseline serum creatinine or more than 20 ml/min per 1.73-m² decrease in estimated GFR (GFR estimation is done on the basis of the four-variable MDRD GFR equation from Levey and colleagues)	Every 4 months
Retinopathy (needs retinal photocoagulation or vitrectomy)	Every year
Peripheral neuropathy (new score of >2.0 on MNSI)	Every year
**Second endpoints**	
**Nephropathy**	
Neph-1: development of microalbuminuria (urine albumin/creatinine ratio, ≥3.4 mg/mmol)	Every year
Neph-2: development of macroalbuminuria (urine albumin/creatinine ratio, ≥33.9 mg/mmol)	Every year
Neph-3: renal failure (initiation of dialysis or end-stage renal disease, renal transplantation, or rise of serum creatinine >291.72 μmol/L)	Every 4 months
**Diabetic eye complications**	
Eye-1: eye surgery for cataract extraction	Every year
Eye-2: three-line change in visual acuity (as measured using LogMAR visual acuity chart)	Every 2 years
Eye-3: severe vision loss (Snellen fraction <20/200)	Every 2 years
**Neuropathy**	
Neuro-1: new loss of vibratory sensation (tested with 128-Hz tuning fork)	Every year
Neuro-2: new loss of ankle jerk during Jendrassik maneuver	Every year
Neuro-3: new loss of light touch (10-*g* force monofilament test)	Every year

CKD, chronic kidney disease; GFR, glomerular filtration rate; MDRD, modification of diet in renal disease; MNSI, Michigan Neuropathy Screening Instrument.

### Patient and Public Involvement

No participants were involved in determining the research questions or outcome measures or in designing or implementing the study. No plans exist to involve patients in knowledge dissemination.

### Statistical Analysis

The relationships between obesity and primary and secondary endpoints were evaluated using the FMI as both a continuous and a categorical variable. Three Cox proportional-hazards regression models were constructed. Model 1 was unadjusted; model 2 was adjusted for age, race, sex, and treatment effect (intensive or standard blood glucose control); and model 3 was adjusted for diabetes duration, proteinuria, current smoking, weekly alcohol consumption, height, and baseline levels of GFR, total cholesterol, low-density lipoprotein cholesterol, high density lipoprotein cholesterol, systolic blood pressure, and hemoglobin A1C, in addition to the four factors adjusted for in model 2. To evaluate FMI as a categorical variable, the patients were divided into sex-specific quartiles of FMI. The first quartile was used as a reference. Tests for linear trends were conducted by treating quartile categories as a continuous variable after assigning the median value for each category (20). To evaluate FMI as a continuous variable, we calculated the hazard ratios (HR) for outcomes per increase in FMI of one standard deviation (SD). Restricted cubic splines with four knots at the fifth, 35th, 65th, and 95th centiles were used to flexibly model the association of FMI or other obesity indicators with our primary endpoints using Cox proportional-hazards models adjusted for factors in model 3. We tested for potential non-linearity by using a likelihood ratio test comparing the model with only a linear term against the model with linear and cubic spline terms. To perform sensitivity analyses, instead of FMI, we used other obesity indicators, such as BMI and WC, and reanalyzed the relationship between obesity and the primary endpoints. Finally, we excluded patients >75 years of age, those with a BMI <18.5 kg/m2, or those with proteinuria. Subgroup and interaction analyses were conducted to explore whether the associations of FMI with the risk of occurrence of the primary outcomes differed by age (<60 and ≥60 years), sex, race, treatment effect (intensive or standard blood glucose control), and smoking.

We further aimed to use discordance analysis to evaluate and compare the effectiveness of FMI, WC, and BMI to predict the risk of microvascular diseases. First, the correction between FMI and other obesity indicators, such as WC and BMI, was computed using Pearson or Spearman rank correlation. Second, we calculated the sex-specific medians for the FMI, WC, and BMI to classify the included participants into two categories based on less than median value or greater than or equal to median values of each obesity indicator. We categorized the participants into four groups according to having a low or a high value of obesity indicators as follows: low/low, high/low, low/high, and high/high. We define a high FMI with WC or BMI being low as discordance or *vice versa*. Finally, Cox proportional-hazards models adjusted for the factors in model 3 were performed on categories of discordant values of (1) FMI *versus* WC, (2) FMI *versus* BMI, and (3) WC *versus* BMI.

All *P*-values were two-sided, and values less than 0.05 were considered statistically significant. All statistical analyses were performed using Stata/SE (version 15.1) and R, version 3.4.3 (R Foundation for Statistical Computing, Vienna, Austria).

### Data and Resource Availability

The data that support the findings of this study are available from BioLINCC, but restrictions apply to the availability of these data, which were used under license for the current study and therefore are not publicly available.

## Results

### Characteristics of Participants

The included participants had a mean age of 62 years and a T2DM duration of median 10 years. A median of six (interquartile range, IQR: 5–7) measurements of the obesity indicators had been performed. The mean (standard deviation, SD) time between baseline FMI and the last measurement was 4.15 ± 1.47 years. The mean (SD) FMI, BMI, WC, and W-t-H values were 12.4 ± 4.1 kg/m^2^, 32.7 ± 5.6 kg/m^2^, 108.0 ± 13.7 cm, and 0.61 ± 0.08, respectively. During the median 5-year (IQR, 4.2-5.7) follow-up period, 6,184 participants developed CKD progression (236 occurrences per 1,000 person-years), 896 participants had retinopathy requiring retinal photocoagulation or vitrectomy (19.8 occurrences per 1,000 person-years), and 3,213 participants developed neuropathy (MNSI >2.0) (6.1 occurrences per 1,000 person-years). The detailed characteristics of the participants are presented in **Table 2**.

**Table 2:** Baseline characteristics of patients with T2DM.

	Men	Women
*N*	6,299	3,952
Obesity-related indicator		
Mean FMI (kg/m^2^)	10.50 ± 2.77	15.50 ± 3.88
Mean BMI (kg/m^2^)	32.13 ± 5.27	33.61 ± 6.07
Mean WC (cm)	109.89 ± 13.36	104.87 ± 13.67
W-t-H		
Age (years)	62.97 ± 6.81	62.56 ± 6.38
Duration of T2DM	10.78 ± 7.57	10.82 ± 7.63
RACE		
Non-white	2,065 (32.78%)	1,793 (45.37%)
White	4,234 (67.22%)	2,159 (54.63%)
Glucose control		
Standard glucose control	3,154 (50.07%)	1,969 (49.82%)
Intensive glucose control	3,145 (49.93%)	1,983 (50.18%)
Baseline CVD	2,586 (41.05%)	1,023 (25.89%)
Live alone	976 (15.49%)	1,102 (27.90%)
Education		
Less than high school	820 (13.03%)	701 (17.75%)
High school graduate	1,543 (24.51%)	1,161 (29.40%)
Some college/technical school	2,063 (32.77%)	1,294 (32.77%)
College graduate or more	1,869 (29.69%)	793 (20.08%)
Proteinuria	1,286 (20.42%)	749 (18.95%)
Neuropathy	1,703 (27.04%)	1,034 (26.16%)
Depression	1,261 (20.02%)	1,160 (29.36%)
Eye diseases	1,881 (29.87%)	1,313 (33.22%)
Current smoker	961 (15.26%)	468 (11.84%)
Alcohol (*n*/week)	1.39 ± 3.25	0.26 ± 0.95
HbA_1C_ (mmol/ml)	8.27 ± 1.04	8.34 ± 1.08
GFR (ml/min/1.73m^2^)	90.94 ± 22.87	91.22 ± 32.85
Lipid (mg/ml)		
Cholesterol	176.71 ± 40.17	193.50 ± 42.19
Triglycerides	193.71 ± 161.30	184.42 ± 127.31
Low-density lipoprotein	101.09 ± 32.39	110.49 ± 35.05
High-density lipoprotein	38.62 ± 9.50	47.07 ± 12.42
SBP (mmHg)	135.63 ± 16.43	137.30 ± 17.80
DBP (mmHg)	74.70 ± 10.61	75.23 ± 10.48
HR (bpm)	71.72 ± 11.86	73.89 ± 11.30

WC, waist circumference; FMI, fat body mass index; BMI, body mass index; W-t-H, waist-to-height; T2DM, type 2 diabetes mellitus; CVD, cardiovascular disease; HbA_1C_, glycosylated hemoglobin; GFR, glomerular filtration rate; SBP, systolic blood pressure; DBP, diastolic blood pressure; HR, heart rate.

### FMI and Microvascular Diseases

The risk of CKD progression and neuropathy (MNSI >2.0) increased with the increased FMI quartiles in the fully adjusted model (model 3). Compared to patients in the lowest FMI quartile, those in the highest quartile were 93 and 26% more likely to develop neuropathy and CKD progression, respectively (**Table 3**). A higher FMI quartile was associated with a numerical increase in retinopathy without statistical significance (HR: 1.17; 95% confidence interval, CI: 0.96–1.43; **Table 3**).

**Table 3:** Hazard ratio (95% CI) of primary endpoints by fat mass index quartiles.

	Incidence rate^a^	Hazard ratio (95%CI)		
		Model 1	Model 2	Model 3
**CKD progression**				
**1**	211	Ref	Ref	Ref
**2**	223	1.05 (0.97–1.13)	1.04 (0.97–1.12)	1.11 (1.03–1.20)
**3**	246	1.14 (1.06–1.23)	1.13 (1.05–1.22)	1.18 (1.09–1.27)
**4**	266	1.22 (1.14–1.31)	1.21 (1.13–1.30)	1.26 (1.16–1.36)
***P*-value for trend**		0.00	0.00	0.00
**Retinopathy** ^b^				
**1**	19	Ref	Ref	Ref
**2**	20	1.08 (0.90–1.31)	1.15 (0.95–1.39)	1.12 (0.92–1.36)
**3**	20	1.04 (0.86–1.25)	1.12 (0.93-1.36)	1.17 (0.96–1.43)
**4**	21	1.09 (0.91–1.31)	1.21 (1.00-1.47)	1.17 (0.96–1.43)
***P*-value for trend**		0.72	0.08	0.16
**Neuropathy** ^c^				
**1**	132	Ref	Ref	Ref
**2**	175	1.31 (1.19–1.45)	1.23 (1.11–1.36)	1.21 (1.09–1.34)
**3**	202	1.51 (1.37–1.67)	1.41 (1.28–1.57)	1.34 (1.21–1.49)
**4**	275	2.00 (1.82–2.22)	1.94 (1.75–2.15)	1.93 (1.74–2.15)
***P*-value for trend**		0.00	0.00	0.00

a Per 1,000 person-years.

b Retinal photocoagulation/vitrectomy.

c New score of >2.0 on Michigan Neuropathy Screening Instrument.

Model 1, unadjusted; model 2, adjusted age, race, sex, and glucose control; model 3, adjusted for age, race, sex, glucose control, duration of diabetes, proteinuria, present smoking, alcohol/week, height, GFR, CHOL, LDL, HDL, SBP, and Hb1AC; CKD, chronic kidney disease; GFR, glomerular filtration rate; CHOL, total cholesterol; LDL, low-density lipoprotein; HDL, high-density lipoprotein; SBP, systolic blood pressure; HbA1C, hemoglobin A1C.

**Figure 1:**
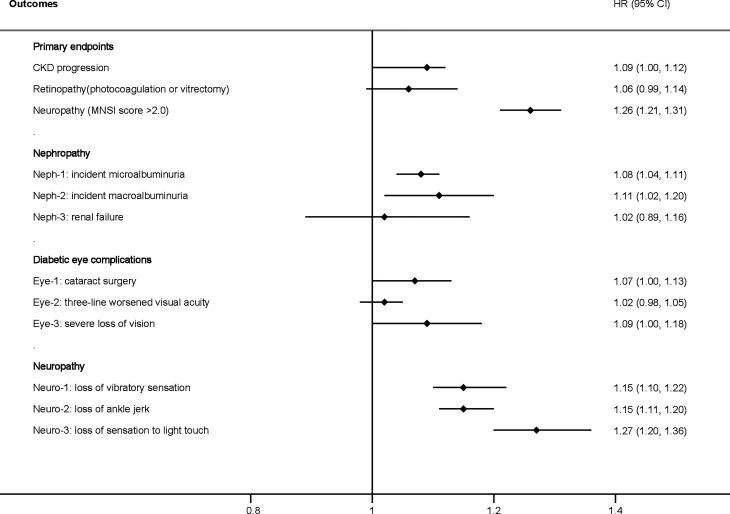
Hazard ratio per one standard deviation increase in the FMI for the primary and secondary endpoints. Adjusted for model 3, including age, race, sex, treatment effect, duration of diabetes, proteinuria, current smoking, weekly alcohol consumption, height, glomerular filtration rate, total cholesterol, low-density lipoprotein cholesterol, high-density lipoprotein cholesterol, systolic blood pressure, and hemoglobin A1C. CKD, chronic kidney disease; HR, hazard ratio; FMI, fat mass index; MNSI, Michigan Neuropathy Screening Instrument.

When FMI was used as a continuous covariate in the fully adjusted model (model 3), each one SD increase in FMI (4.0 kg/m^2^) increased the risk of CKD progression (HR: 1.09, 95% CI: 1.00–1.12) and neuropathy (MNSI >2.0) (HR: 1.26, 95% CI: 1.21–1.31), with a numerical increase in retinopathy (HR: 1.06, 95% CI: 0.99–1.14) without statistical significance (**Figure 1**). A higher FMI was associated with nephropathy, including ESRD (HR: 1.08, 95% CI: 1.04–1.11), microalbuminuria (HR: 1.08, 95% CI: 1.04–1.11), macroalbuminuria (HR: 1.11, 95% CI: 1.02–1.20), and neuropathy, including the loss of vibratory sensation (HR: 1.15, 95% CI: 1.10–1.22), ankle jerk (HR: 1.15, 95% CI: 1.11–1.20), and sensation to light touch (HR: 1.27, 95% CI: 1.20–1.36) (**Figure 1**). Patients with a higher FMI had a marginally increased risk of diabetic eye complications, including cataract surgery (HR: 1.07, 95% CI: 1.00–1.13), worsened three-line visual acuity (HR: 1.02, 95% CI: 0.98–1.05), and severe loss of vision (HR: 1.09, 95% CI: 1.00–1.18) (**Figure 1**).

**Figure 2:**
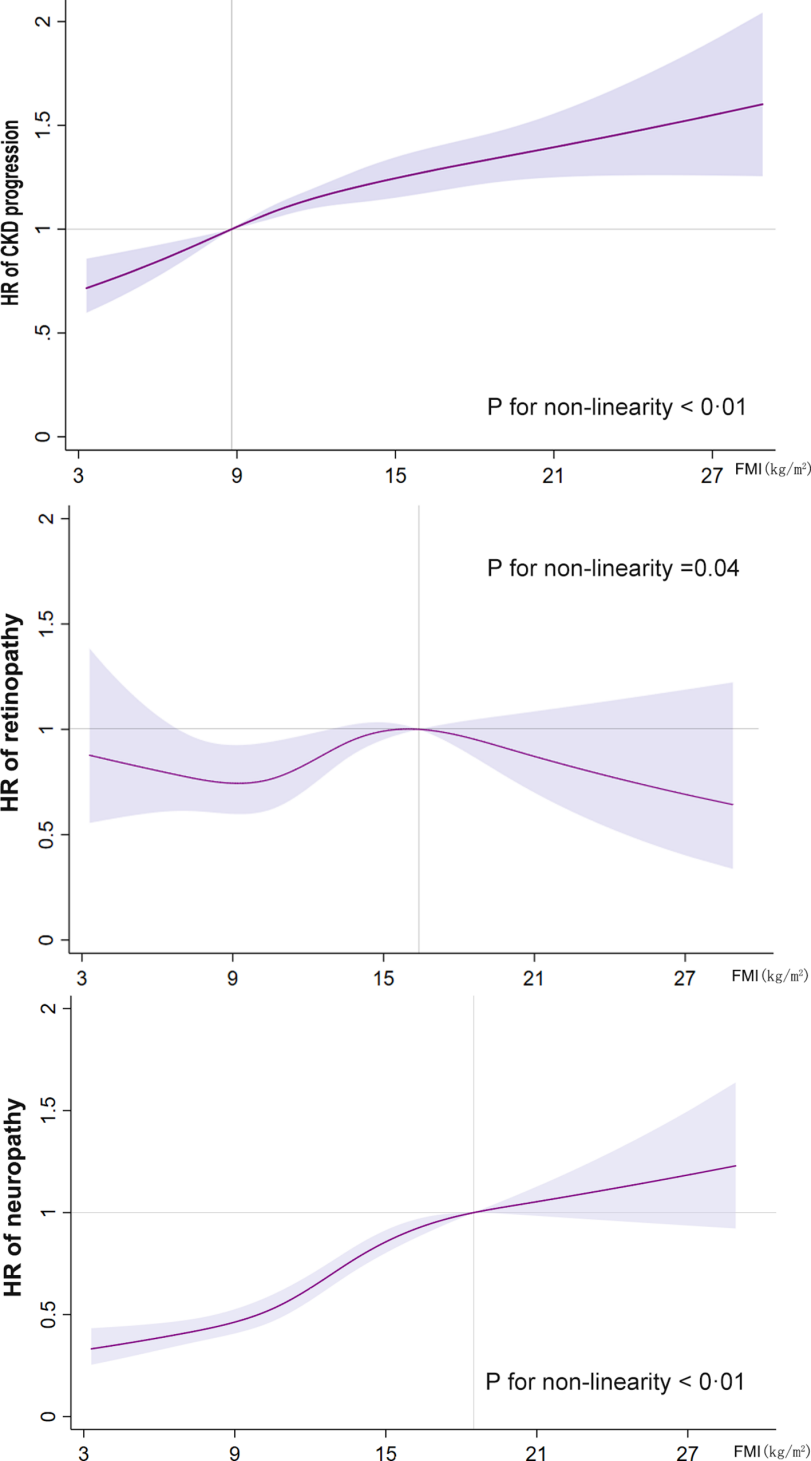
Restricted cubic spline analysis of the fat mass index for the estimation of the risk of the primary endpoints after adjusting for multivariate covariates. Hazard ratios are indicated by solid lines and the 95% confidence intervals by shaded areas. The reference point is the inflection point for each FMI (8.8 kg/m^2^ for CKD progression, 16.4 kg/m^2^ for retinopathy, and 18.5 kg/m^2^ for neuropathy), with knots placed at the fifth, 35th, 65th, and 95th percentiles of each FMI distribution. The hazard ratios shown are adjusted for model 3, including age, race, sex, treatment effect, diabetes duration, proteinuria, current smoking, weekly alcohol consumption, height, glomerular filtration rate, total cholesterol, low-density lipoprotein cholesterol, high-density lipoprotein cholesterol, systolic blood pressure, and hemoglobin A1C. CKD, chronic kidney disease; FMI, fat mass index; HR, hazard ratio.

In **Figure 2**, we used restricted cubic splines to flexibly model and visualize the relationship between FMI and the primary endpoints. The risk of CKD progression increased rapidly, peaked at approximately 8.8 kg/m^2^, and then remained relatively unchanged (for non-linearity, *p <*0.01). However, both the lower and higher ranges of FMI showed a higher risk of CKD progression. Regarding the inverted U-shaped relationship between FMI and retinopathy, the plot showed a substantial increase in the risk of retinopathy within the lower range of FMI, reaching the highest risk at around 16.4 kg/m^2^ and decreasing thereafter (for non-linearity, *p <*0.04). The risk of neuropathy increased sharply to approximately 18.5 kg/m^2^ and then leveled off (for non-linearity, *p <*0.01). A “plateau phenomenon” was found in the relationship between FMI and the risk of CKD progression or neuropathy (MNSI >2.0).

### Sensitivity and Subgroup Analysis

**Figure 3:**
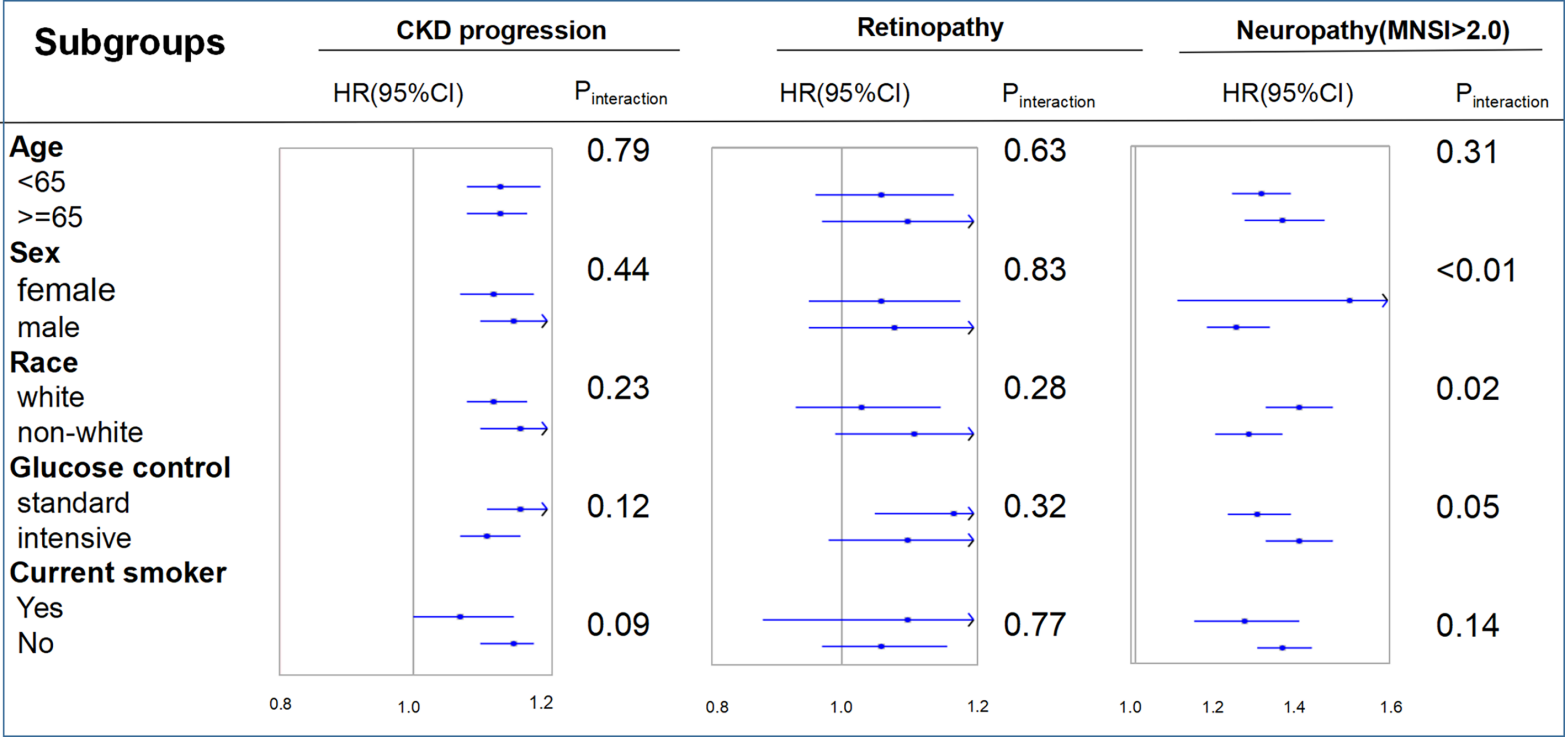
Hazard ratios per one standard deviation increase in the fat mass index for the primary endpoints. Each stratification was adjusted for all factors in model 3 (age, race, sex, treatment effect, diabetes duration, proteinuria, current smoking, weekly alcohol consumption, height, glomerular filtration rate, total cholesterol, low-density lipoprotein cholesterol, high-density lipoprotein cholesterol, systolic blood pressure, and hemoglobin A1C), except for the stratification factor itself. CKD, chronic kidney disease; FMI, fat mass index; MNSI, Michigan Neuropathy Screening Instrument.

**Figure 3** shows the interaction and subgroup analysis results. No factor played an interactive role in the association between FMI and CKD progression or retinopathy. However, glucose control played an important role in the relationship between FMI and neuropathy (MNSI >2.0), suggesting a stronger effect of FMI on the risk of neuropathy (MNSI >2.0) in the intensive glucose control subgroup. Similar stronger effects were also found in female and white participants. When other indicators of obesity were used, such as BMI and WC, the primary and secondary endpoints remained unchanged (**Supplementary Figures S1**, **S2** and **Supplementary Tables S2**, **S3**). The observed associations of obesity (FMI) with primary and secondary endpoints were unchanged after we excluded participants >75 years of age, those with a BMI <18.5 kg/m^2^, or those with proteinuria (**Supplementary Tables S4**–**S6**).

### Discordant Analysis

**Figure 4:**
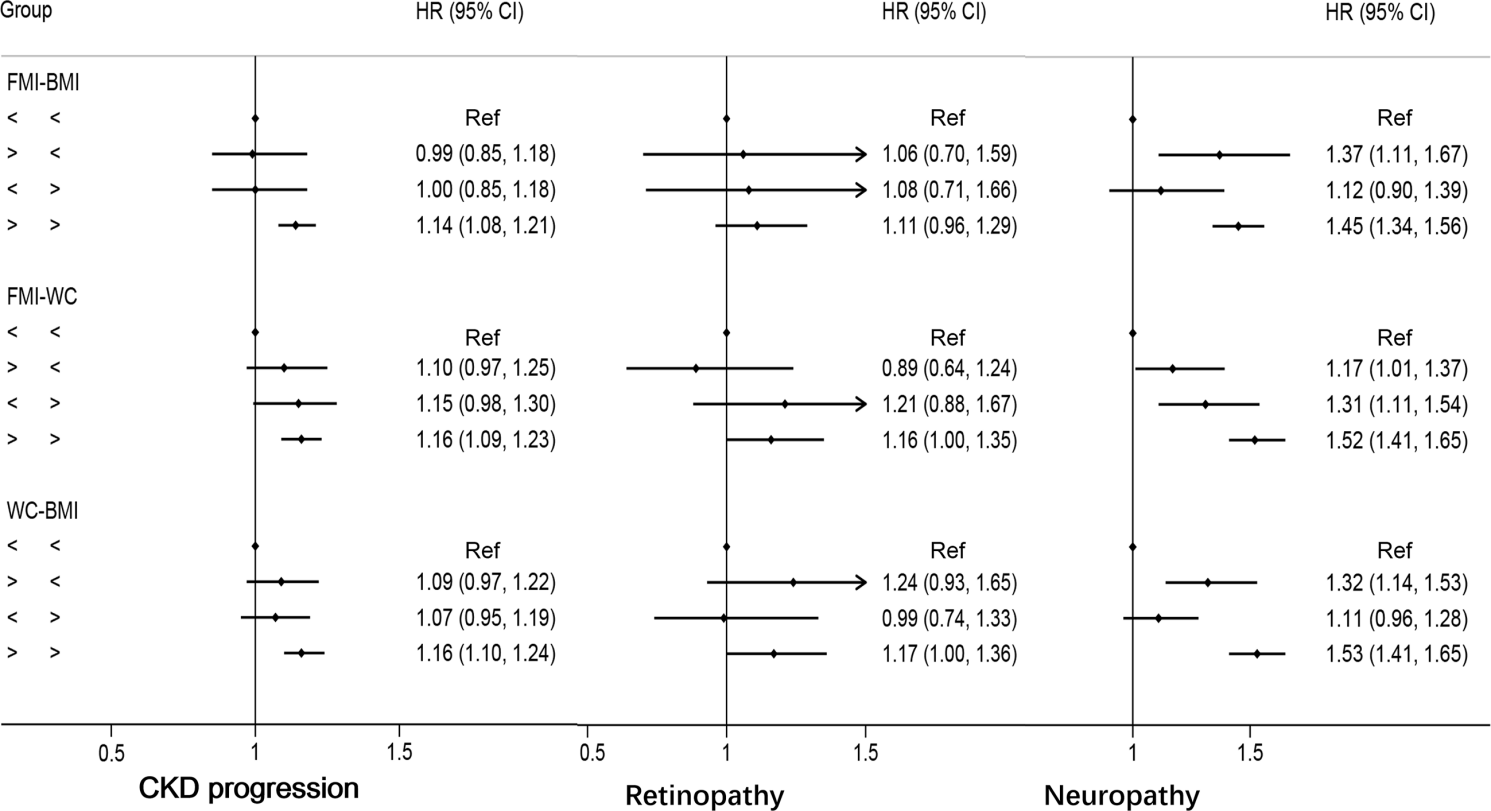
Multivariable-adjusted hazard ratios of primary endpoints by discordant *versus* concordant categories of fat mass index, body mass index, and waist circumference in type 2 diabetes mellitus patients. Adjusted for model 3: age, race, sex, glucose control, diabetes duration, proteinuria, current smoking, weekly alcohol consumption, height, glomerular filtration rate, total cholesterol, low density lipoprotein cholesterol, high density lipoprotein cholesterol, systolic blood pressure, and hemoglobin A1C. HR, hazard ratios; CI, confidence interval; CKD, chronic kidney disease.

Discordant FMI above the median with BMI below was not associated with an increased risk of CKD progression (HR: 0.99, 95%CI: 0.85–1.18) compared with concordant FMI and BMI below the medians; similar results were found for discordant FMI above the median and WC below (HR: 1.10, 95%CI: 0.97–1.25) and discordant WC above the median with BMI below (HR:1.09, 95%CI: 0.97–1.22, **Figure 4**). No clear trend was observed for discordance between FMI, WC, and BMI, with risk for retinopathy only being marginally higher if both are above the median (**Figure 4**). Discordant FMI above the medians and BMI below the medians presented an HR of 1.37 (95%CI: 1.11–1.67) for neuropathy, compared with concordant WC and BMI below the medians; similar results were found for discordant WC above the median with BMI below (HR:1.32, 95%CI: 1.14–1.53, **Figure 4**) compared with concordant WC and BMI below the means. Discordant FMI above the median with WC below the median yielded a HR of 1.17 (95% CI: 1.01 to 1.37), while discordant low FMI with WC above the median was also associated with a higher risk of neuropathy (HR: 1.31; 95% CI: 1.11–1.54, **Figure 4**).

## Discussion

This study is a *post-hoc* analysis of 10,251 patients with T2DM who either had CVD or had a high risk of developing CVD. First, we find that obesity was associated with an increased risk of microalbuminuria, macroalbuminuria, deterioration of renal function, and neuropathy. Second, obese patients with T2DM had a marginally increased risk of retinopathy but did not have an increased risk of ESRD. Third, a stronger effect of FMI on the risk of neuropathy (MNSI >2.0) was found in intensive glucose control, female, or white race subgroup. Fourth, discordance analysis demonstrated that FMI or WC was a more accurate indicator of obesity-related risk of neuropathy compared with BMI.

Consistent with the current study, previous studies have pointed out an increased incidence of microalbuminuria, macroalbuminuria, deterioration of renal function, and neuropathy in T2DM patients who are obese (10, 21). The association of obesity with the deterioration of renal function or neuropathy got stronger as the severity of obesity increased, which indicates suggesting a dose–response relationship. Furthermore, recent studies have found that gastric bypass surgery results in robust, beneficial renal outcomes in obese patients with T2DM (22–24). Together these findings suggest that obese patients with T2DM have a higher risk of deterioration of renal function and therefore should focus on weight control. However, we did not find that obese patients with T2DM had a higher risk of ESRD than their non-obese counterparts. This paradoxical result may be due to the short-term follow-up period, which did not allow us to examine the detrimental effects of obesity on renal failure.

We did not find an exact relationship between obesity and the risk of retinopathy. A previous study has found that weight control may be less useful in T2DM patients with retinopathy. Although bariatric surgery reduces the risk of nephropathy and neuropathy significantly in T2DM patients, it did not reduce the risk of retinopathy, which was consistent with our present study (22–25). Another reason that we did not find an exact relationship between obesity and the risk of retinopathy is the lower incidence of retinopathy compared with neuropathy and CKD progression. Further research is needed regarding the association between obesity and the risk of retinopathy in T2DM patients.

Consistent with previous studies, obesity was associated with an increased risk of neuropathy. However, our present study extends the knowledge on the relationship between obesity and risk of neuropathy. FMI and WC are a more accurate indicators of obesity-related risk of neuropathy compared with BMI. However, no studies have performed discordance analyses on T2DM patients as done in our analyses. In cohorts with T2DM, discordance analyses also favor FMI and WC in identifying individuals with obesity-related risk of neuropathy. FMI and WC reflect different aspects of obesity with added effect on each other. Therefore, when we evaluate the risk of neuropathy, FMI or WC may be more appropriate than BMI. A subgroup among obese patients with lower FM/WC, called metabolically healthy obesity, has recently been recognized, which does not express metabolic disorders and cardiovascular risk factors. A subgroup of normal-weight patients with higher FM/WC has metabolic disorders and cardiovascular risk factors. Importantly, abdominal obesity is a modifiable risk factor, and thus the question arises whether weight reduction in individuals who are overweight or with obesity by interventions, such as lifestyle modification or bariatric surgery, have an influence on the prevention of neuropathy.

### Limitations

This study has several limitations. First, the FM that we used was calculated with equations rather than the true FM, which is measured by dual-energy radiographic examination. However, the prediction equations, including factors such as age, sex, height, weight, and WC, showed a high predictive ability for FM and have been widely used in epidemiological studies. Furthermore, we obtained similar results using different anthropometric indicators suggesting a robust association between obesity and microvascular diseases. Second, the short follow-up duration might explain why we could not find out whether obese patients with T2DM had a high risk of renal failure. Finally, this study only included patients from the United States and Canada; hence, these results may not apply to other populations with different body habitus.

## Conclusion

Obesity is associated with microalbuminuria, macroalbuminuria, neuropathy, and deterioration of renal function in participants with T2DM. Further randomized trials are needed to test whether obesity control can improve the outcomes of T2DM participants with CKD or neuropathy. The FMI and WC are more useful in identifying individuals with obesity-related risk of neuropathy compared with BMI in T2DM patients.

## Data Availability Statement

Data are available from the Biologic Specimen and Data Repository Information Coordinating Center (BioLINCC).

## Ethics Statement

This is a *post-hoc* analysis of ACCORD study. No participants were involved in setting the research questions or outcome measures or in the design and implementation of the study. No plans exist to involve patients in dissemination.

## Author Contributions

ZX designed the study and provided methodological expertise. ZX and SG drafted the manuscript. HZ and CL revised the manuscript. ZX is the guarantor of this work and, as such, had full access to all the data in the study and takes responsibility for the integrity of the data and the accuracy of the data analysis. All authors contributed to the article and approved the submitted version.

## Funding

This work was supported in part by the National Science Foundation of China project 82000298, the Natural Science Foundation of Hunan Province 2021JJ40883 to ZX, the Natural Science Foundation of Hunan Province 2019JJ40451 to HZ, and the Natural Science Foundation of Hunan Province 2021JJ40850 to CL.

## Conflict of Interest

The authors declare that the research was conducted in the absence of any commercial or financial relationships that could be construed as a potential conflict of interest.

## Publisher’s Note

All claims expressed in this article are solely those of the authors and do not necessarily represent those of their affiliated organizations, or those of the publisher, the editors and the reviewers. Any product that may be evaluated in this article, or claim that may be made by its manufacturer, is not guaranteed or endorsed by the publisher.
