# Supplementary material for: Association Between Obesity and Microvascular Diseases in Patients With Type 2 Diabetes Mellitus
Source: Front Endocrinol (Lausanne). 2021 Oct 26;12:719515. doi: 10.3389/fendo.2021.719515 (PMC8576347; doi:10.3389/fendo.2021.719515)
Supplement: Supplementary file 1 [file DataSheet_1.docx]

**Supplementary file**

**sTable 1：**Anthropometric prediction equations for fat mass

**sTable 2:** Spearman correlations among obesity indicator

**sTable 3：**Hazard ratio (95% CI) of primary endpoints according to BMI quartiles.

**sTable 4**：Hazard ratio (95% CI) of primary endpoints according to WC quartiles.

**sTable 5:** Association between FMI quartiles and primary endpoints by excluding patients with age >75 years

**sTable 6:** Association between FMI quartiles and primary endpoints by excluding patients with BMI <18.5kg/m^2^

**sTable 7**: Association between FMI quartiles and primary endpoints by excluding patients with proteinuria

**sFigure 1:** The relationship between BMI and primary and second endpoints

**sFigure 2:** The relationship between WC and primary and second endpoints

**sTable 1：Anthropometric prediction equations for lean body mass and fat mass**

| Fat mass |  | R^2^ | SEE(kg) |
| --- | --- | --- | --- |
|  |  |  |  |
| Men | -18.592–0.009*age(years)–0.080*height(cm)+0.226*weight(kg)+ 0.387*waist(cm)+0.080*Mexican–0.188*Hispanic–0.483*Black + 1.050*Other ethnicity | 0.90 | 2.60 |
| Women | 11.817+0.041*age(years)–0.199*height(cm)+0.610*weight(kg)+ 0.044*waist(cm)+0.388*Mexican+0.073*Hispanic–1.187*Black+0.325*Other ethnicity | 0.93 | 2.44 |

**sTable 2：Hazard ratio (95% CI) of primary endpoints according to BMI quartiles.**

|  | Incidence rate * | Hazard ratio(95%CI) | | | |
| --- | --- | --- | --- | --- | --- |
| CKD progression |  |  |  | | |
| 1 | 213 | Ref | Ref | Ref |  |
| 2 | 223 | 1.04(0.97–1.12) | 1.03(0.96–1.11) | 1.09(1.01–1.18) |  |
| 3 | 248 | 1.13(1.06–1.22) | 1.12(1.04–1.20) | 1.10(1.10–1.28) |  |
| 4 | 262 | 1.19(0.11–1.28) | 1.17(1.09–1.26) | 1.21(1.12–1.31) |  |
| P value for trend |  | 0.00 | 0.00 | 0.00 |  |
| Retinopathy# |  |  |  |  |  |
| 1 | 18.3 | Ref | Ref | Ref |  |
| 2 | 20.5 | 1.12(0.93–1.35) | 1.18(0.97–1.42) | 1.18(0.98–1.44) |  |
| 3 | 19.8 | 1.08(0.89–1.30) | 1.16(0.96-1.40) | 1.21(0.99–1.48) |  |
| 4 | 20.7 | 1.12(0.93–1.36) | 1.24(1.02-1.49) | 1.22(0.99–1.49) |  |
| P value for trend |  | 0.30 | 0.05 | 0.08 |  |
| Neuropathy& |  |  |  |  | |
| 1 | 138 | Ref | Ref | Ref | |
| 2 | 175 | 1.25(1.13–1.38) | 1.19(1.08–1.32) | 1.17(1.06–1.30) | |
| 3 | 200 | 1.42(1.29–1.57) | 1.38(1.25-1.53) | 1.31(1.18–1.46) | |
| 4 | 263 | 1.83(1.66–2.02) | 1.86(1.68-2.06) | 1.82(1.64–2.02) | |
| P value for trend |  | 0.00 | 0.00 | 0.00 | |

*Per 1000 person-years; # retinal photocoagulation/ vitrectomy; &New score of >2·0 on MNSI

Model 1: unadjusted; Model 2: adjusted age, race, sex glucose control; Model 3: adjusted for age, race, sex, glucose control, diabetes duration, proteinuria, current smoking, weekly alcohol consumption, height, glomerular filtration rate, total cholesterol, low density lipoprotein cholesterol, high density lipoprotein cholesterol, systolic blood pressure, and hemoglobin A1C

FMI= fat mass index, CKD=chronic kidney disease

**sTable 3：Hazard ratio (95% CI) of primary endpoints according to WC quartiles.**

|  | Incidence rate * | Hazard ratio(95%CI) | | | |
| --- | --- | --- | --- | --- | --- |
| CKD progression |  |  |  | | |
| 1 | 203 | Ref | Ref | Ref |  |
| 2 | 234 | 1.13(1.05–1.22) | 1.13(1.05–1.22) | 1.21(1.12–1.30) |  |
| 3 | 246 | 1.17(1.09–1.26) | 1.17(1.09–1.25) | 1.26(1.16–1.36) |  |
| 4 | 263 | 1.25(1.16–1.34) | 1.24(1.15–1.33) | 1.32(1.22–1.43) |  |
| P value for trend |  | 0.00 | 0.00 | 0.00 |  |
| Retinopathy# |  |  |  |  |  |
| 1 | 18.9 | Ref | Ref | Ref |  |
| 2 | 19.6 | 1.03(0.85–1.24) | 1.07(0.89–1.30) | 1.14(0.94–1.39) |  |
| 3 | 20.1 | 1.06(0.88–1.27) | 1.13(0.94-1.37) | 1.26(1.03–1.53) |  |
| 4 | 20.7 | 1.09(0.90–1.31) | 1.19(0.99-1.45) | 1.26(1.03–1.55) |  |
| P value for trend |  | 0.35 | 0.06 | 0.02 |  |
| Neuropathy& |  |  |  |  | |
| 1 | 130 | Ref | Ref | Ref | |
| 2 | 172 | 1.32(1.19–1.46) | 1.24(1.12–1.37) | 1.18(1.07–1.32) | |
| 3 | 215 | 1.62(1.47–1.79) | 1.52(1.37-1.68) | 1.42(1.28–1.57) | |
| 4 | 280 | 2.09(1.89–2.31) | 2.00(1.81-2.22) | 1.89(1.70–2.11) | |
| P value for trend |  | 0.00 | 0.00 | 0.00 | |

*Per 1000 person-years; # retinal photocoagulation/ vitrectomy; &New score of >2·0 on MNSI

Model 1: unadjusted; Model 2: adjusted age, race, sex glucose control; Model 3: adjusted for age, race, sex, glucose control, diabetes duration, proteinuria, current smoking, weekly alcohol consumption, height, glomerular filtration rate, total cholesterol, low density lipoprotein cholesterol, high density lipoprotein cholesterol, systolic blood pressure, and hemoglobin A1C

FMI= fat mass index, CKD=chronic kidney disease

**sTable 4: Association between FMI quartiles and primary endpoints by excluding patients with age >75 years**

|  | Hazard ratio(95%CI) | | | |
| --- | --- | --- | --- | --- |
| CKD progression |  |  | | |
| 1 | Ref | Ref | Ref |  |
| 2 | 1.04(0.97–1.13) | 1.04(0.97–1.13) | 1.11(1.03–1.20) |  |
| 3 | 1.13(1.06–1.22) | 1.12(1.04–1.21) | 1.16(1.08–1.26) |  |
| 4 | 1.19(1.11–1.28) | 1.20(1.11–1.29) | 1.25(1.15–1.35) |  |
| P value for trend | 0.00 | 0.00 | 0.00 |  |
| Retinopathy# |  |  |  |  |
| 1 | Ref | Ref | Ref |  |
| 2 | 1.06(0.88–1.29) | 1.13(0.93–1.37) | 1.11(0.91–1.35) |  |
| 3 | 1.01(0.84–1.23) | 1.10(0.90-1.34) | 1.15(0.94–1.41) |  |
| 4 | 1.08(0.89–1.30) | 1.21(1.00-1.48) | 1.17(0.95–1.44) |  |
| P value for trend | 0.55 | 0.05 | 0.13 |  |
| Neuropathy& |  |  |  | |
| 1 | Ref | Ref | Ref | |
| 2 | 1.35(1.22–1.50) | 1.24(1.12–1.38) | 1.23(1.10–1.36) | |
| 3 | 1.55(1.40–1.72) | 1.42(1.28-1.58) | 1.35(1.21–1.50) | |
| 4 | 2.09(1.89–2.31) | 1.98(1.78-2.20) | 1.97(1.76–2.19) | |
| P value for trend | 0.00 | 0.00 | 0.00 | |

# retinal photocoagulation/ vitrectomy; &New score of >2·0 on MNSI

Model 1: unadjusted; Model 2: adjusted age, race, sex glucose control; Model 3: adjusted for age, race, sex, glucose control, diabetes duration, proteinuria, current smoking, weekly alcohol consumption, height, glomerular filtration rate, total cholesterol, low density lipoprotein cholesterol, high density lipoprotein cholesterol, systolic blood pressure, and hemoglobin A1C

FMI= fat mass index, CKD=chronic kidney disease

**sTable 5: Association between FMI quartiles and primary endpoints by excluding patients with BMI <18.5kg/m^2^**

|  | Hazard ratio(95%CI) | | | |
| --- | --- | --- | --- | --- |
| CKD progression | . |  | | |
| 1 | Ref | Ref | Ref |  |
| 2 | 1.04(0.97–1.13 | 1.04(0.97–1.12) | 1.08(0.99–1.17) |  |
| 3 | 1.14(1.06–1.22) | 1.13(1.05–1.22) | 1.15(1.05–1.25) |  |
| 4 | 1.22(1.14–1.31) | 1.21(1.13–1.30) | 1.26(1.16–1.38) |  |
| P value for trend | 0.00 | 0.00 | 0.00 |  |
| Retinopathy# |  |  |  |  |
| 1 | Ref | Ref | Ref |  |
| 2 | 1.08(0.90–1.31) | 1.15(0.95–1.39) | 1.12(0.92–1.36) |  |
| 3 | 1.04(0.86–1.25) | 1.12(0.93-1.36) | 1.17(0.96–1.43) |  |
| 4 | 1.09(0.91–1.31) | 1.21(1.00-1.47) | 1.17(0.96–1.43) |  |
| P value for trend | 0.30 | 0.05 | 0.14 |  |
| Neuropathy& |  |  |  | |
| 1 | Ref | Ref | Ref | |
| 2 | 1.31(1.19–1.45) | 1.23(1.11–1.36) | 1.21(1.09–1.34) | |
| 3 | 1.51(1.37–1.67) | 1.41(1.28-1.57) | 1.34(1.21–1.49) | |
| 4 | 2.01(1.82–2.22) | 1.94(1.75-2.15) | 1.93(1.74–2.15) | |
| P value for trend | 0.00 | 0.00 | 0.00 | |

# retinal photocoagulation/ vitrectomy; &New score of >2·0 on MNSI

Model 1: unadjusted; Model 2: adjusted age, race, sex glucose control; Model 3: adjusted for age, race, sex, glucose control, diabetes duration, proteinuria, current smoking, weekly alcohol consumption, height, glomerular filtration rate, total cholesterol, low density lipoprotein cholesterol, high density lipoprotein cholesterol, systolic blood pressure, and hemoglobin A1C

FMI= fat mass index, BMI=body mass index, CKD=chronic kidney disease

**sTable 6: Association between FMI quartiles and primary endpoints by excluding patients with proteinuria**

|  | Hazard ratio(95%CI) | | | |
| --- | --- | --- | --- | --- |
| CKD progression |  |  | | |
| 1 | Ref | Ref | Ref |  |
| 2 | 1.01(0.94–1.10) | 1.01(0.93–1.10) | 1.08(0.99–1.17) |  |
| 3 | 1.11(1.03–1.21) | 1.10(1.02–1.19) | 1.15(1.05–1.25) |  |
| 4 | 1.21(1.12–1.31) | 1.20(1.10–1.30) | 1.26(1.16–1.38) |  |
| P value for trend | 0.00 | 0.00 | 0.00 |  |
| Retinopathy# |  |  |  |  |
| 1 | Ref | Ref | Ref |  |
| 2 | 1.06(0.86–1.32) | 1.14(0.91–1.42) | 1.12(0.89–1.41) |  |
| 3 | 1.04(0.84–1.30) | 1.14(0.91-1.42) | 1.19(0.95–1.50) |  |
| 4 | 1.03(0.83–1.29) | 1.17(0.93-1.47) | 1.18(0.93–1.50) |  |
| P value for trend | 0.72 | 0.23 | 0.24 |  |
| Neuropathy& |  |  |  | |
| 1 | Ref | Ref | Ref | |
| 2 | 1.35(1.21–1.51) | 1.26(1.12–1.41) | 1.23(1.10–1.38) | |
| 3 | 1.58(1.41–1.76) | 1.48(1.32-1.65) | 1.41(1.25–1.58) | |
| 4 | 2.11(1.89–2.36) | 2.03(1.81-2.28) | 2.05(1.82–2.30) | |
| P value for trend | 0.00 | 0.00 | 0.00 | |

# retinal photocoagulation/ vitrectomy; &New score of >2·0 on MNSI

Model 1: unadjusted; Model 2: adjusted age, race, sex glucose control; Model 3: adjusted for age, race, sex, glucose control, diabetes duration, proteinuria, current smoking, weekly alcohol consumption, height, glomerular filtration rate, total cholesterol, low density lipoprotein cholesterol, high density lipoprotein cholesterol, systolic blood pressure, and hemoglobin A1C

FMI= fat mass index, CKD=chronic kidney disease


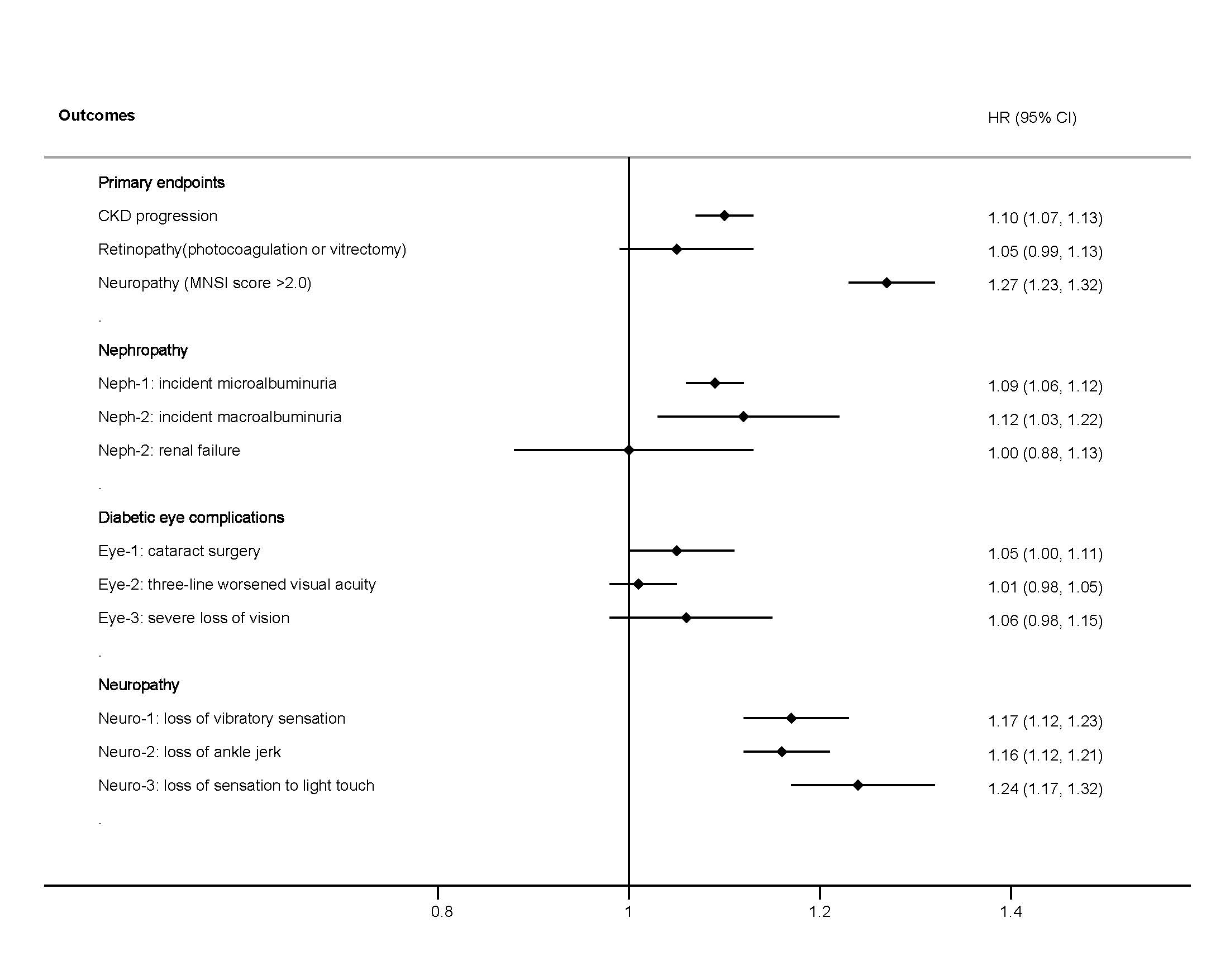


**sFigure 1: the relationship between BMI and primary and second endpoints**

adjusted for Model 3: adjusted for age, race, sex, glucose control, diabetes duration, proteinuria, current smoking, weekly alcohol consumption, height, glomerular filtration rate, total cholesterol, low density lipoprotein cholesterol, high density lipoprotein cholesterol, systolic blood pressure, and hemoglobin A1C

**
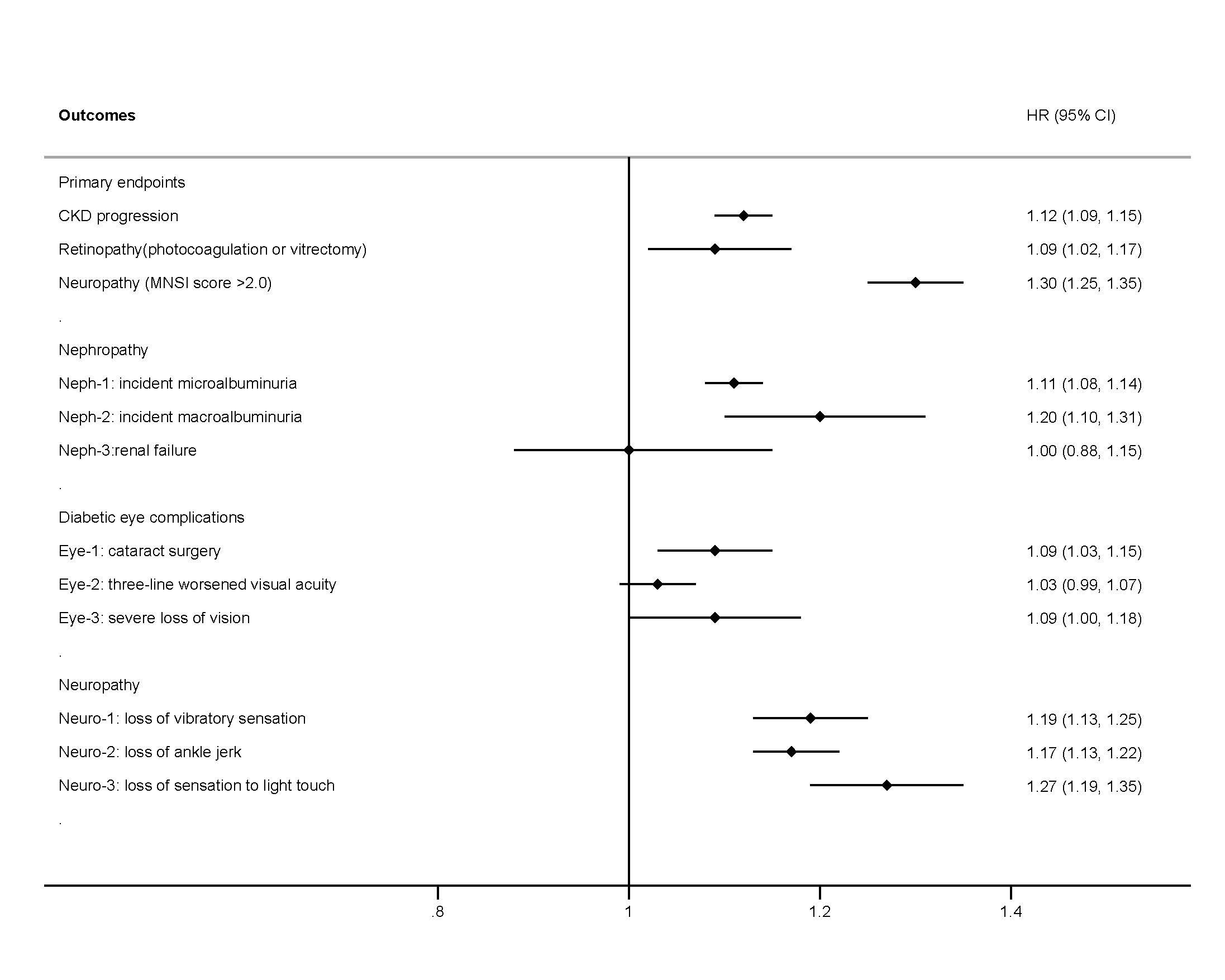
**

**sFigure 2: the relationship between WC and primary and second endpoints**

adjusted for Model 3: adjusted for age, race, sex, glucose control, diabetes duration, proteinuria, current smoking, weekly alcohol consumption, height, glomerular filtration rate, total cholesterol, low density lipoprotein cholesterol, high density lipoprotein cholesterol, systolic blood pressure, and hemoglobin A1C

**
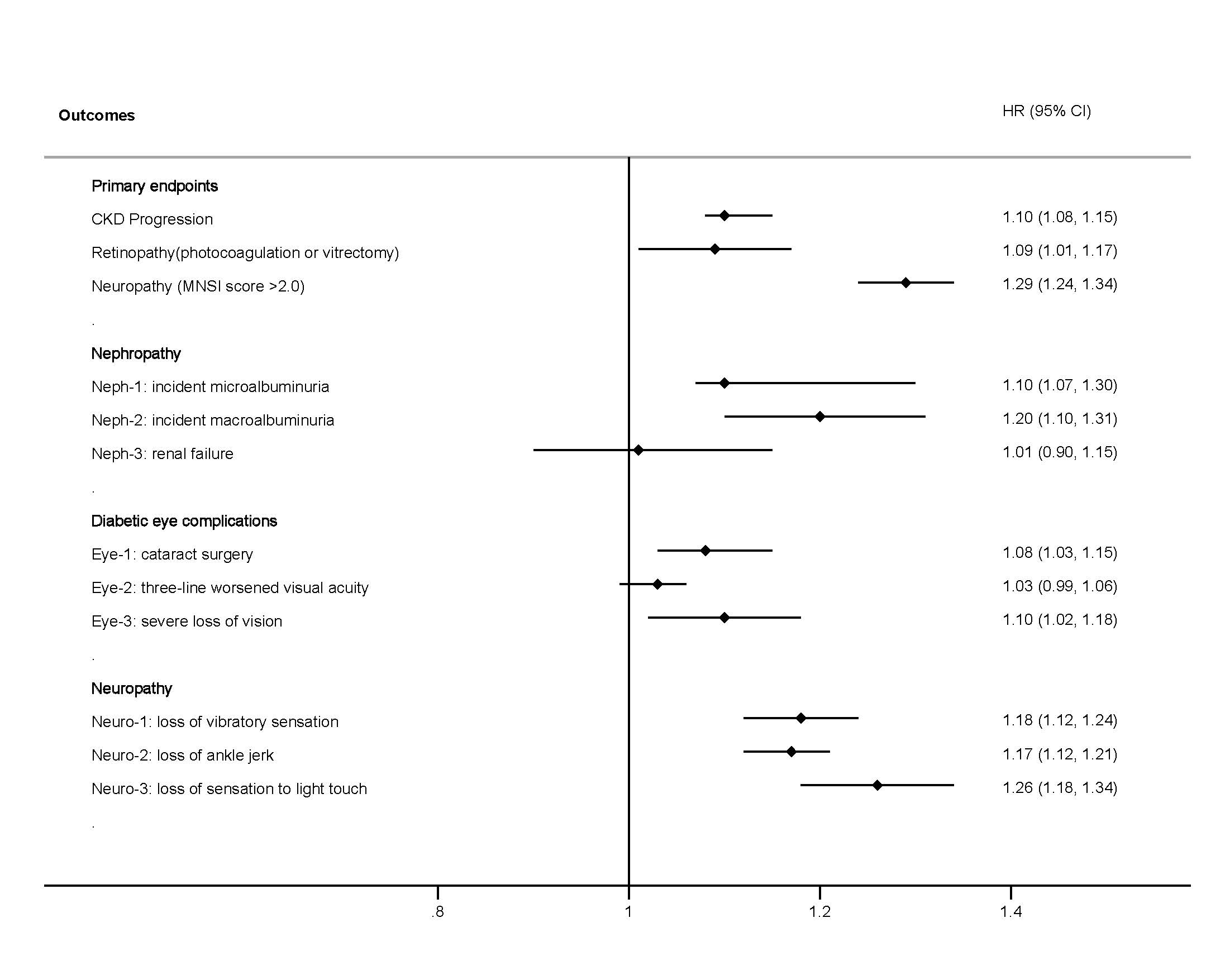
**

**sFigure 3: the relationship between W-t-H and primary and second endpoints**

adjusted for Model 3: adjusted for age, race, sex, glucose control, diabetes duration, proteinuria, current smoking, weekly alcohol consumption, height, glomerular filtration rate, total cholesterol, low density lipoprotein cholesterol, high density lipoprotein cholesterol, systolic blood pressure, and hemoglobin A1C
